# Draft Genome Sequence of the Photoheterotrophic *Chloracidobacterium thermophilum* Strain OC1 Found in a Mat at Ojo Caliente

**DOI:** 10.1128/genomeA.01570-15

**Published:** 2016-02-18

**Authors:** Patrick C. Hallenbeck, Melanie Grogger, Megan Mraz, Donald Veverka

**Affiliations:** Department of Biology, Life Sciences Research Center, U.S. Air Force Academy, Colorado Springs, Colorado, USA

## Abstract

Metagenomics of an enrichment culture from a New Mexico hot spring allowed the description of a draft genome of a *Chloracidobacterium thermophilum* strain for the first time outside Yellowstone National Park with a surprisingly high degree of identity with the type strain.

## GENOME ANNOUNCEMENT

Relatively recently, a novel photoheterotroph found in Octopus Hot Springs, Yellowstone National Park (YNP), *Chloracidobacterium thermophilum*, was described (1, 2). This finding extended chlorophyll-dependent phototrophy to a sixth, little known bacterial phylum, *Acidobacter*, and showed that the organization of bacterial photosynthesis in chlorosomes, supramolecular photosynthetic structures allowing for very efficient photosynthesis at low light intensities, extends to a third bacterial group (3, 4). A wealth of information concerning molecular details of its photosynthetic system have been obtained, including its homodimeric type I reaction center (RCI) (5–8). A detailed molecular understanding of the photosynthetic system of this organism has helped inform the ongoing debate on the evolution and origin of oxygenic photosynthesis (9, 10).

Interesting insights were gained 3 years ago when the complete genome sequence of *C. thermophilum* was determined, even though it was still growing in a mixed culture (11). Among other things, this information helped to define the medium supplements necessary to permit the axenic growth of this fastidious organism (12), which then permitted complete strain characterization and taxonomic acceptance (13). Although until now this organism has been described in genomic detail from samples obtained at Octopus Hot Springs, YNP, an ecological study showed that, based on 16S rRNA analysis, *C. thermophilum* is abundant and widespread among mats at different YNP hot springs (14).

However, until now the presence of *C. thermophilum* outside YNP has not been reported even though it may represent a widespread organism of ecological importance in hot springs mats. Thus, it would be important to describe in detail strains of this organism that might be found elsewhere. Here we report on the draft genome of strain OC1, found at a thermal source in New Mexico. The OC1 draft genome was 62.4% GC and consisted of 3,600,358 bp. Surprisingly, OC1 shows a great deal of similarity with the genome of the *C. thermophilum* type strain, with a high degree of identity with both SSU RNA (1404/1424, 99%) and LSU RNA (2853/2900, 98%). The draft genome consisted of 3,062 coding sequences and 49 RNAs, as determined by RAST and SEED (15, 16). Strikingly, 1,215 of the predicted OC1 coding sequences shared 95% or greater identity with those of the type strain.

Genomic DNA was isolated from an enrichment culture of a sample obtained at a thermal source at Ojo Caliente, New Mexico. The library was prepared using a Nextera DNA sample preparation kit (Illumina) following the manufacturer’s user guide, with subsequent simultaneous fragmentation and addition of adapter sequences by a limited-cycle (5 cycles) PCR. The final library concentration (2.28 ng/µL) was measured using the Qubit dsDNA HS assay kit (Life Technologies), and the average library size (887 bp) was determined using the Agilent 2100 Bioanalyzer (Agilent Technologies). The library was sequenced by using a 600 Cycles v3 reagent kit (Illumina) in MiSeq (Illumina). Assembly was performed by MR DNA (Shallowater, TX) using NGEN (DNAstar) as the primary assembly method, followed by manual optimization.

### Nucleotide sequence accession numbers.

This whole-genome shotgun project has been deposited at DDBJ/EMBL/GenBank under the accession number LMXM00000000. The version described in this paper is version LMXM01000000
